# Annexin-A6 in Membrane Repair of Human Skeletal Muscle Cell: A Role in the Cap Subdomain

**DOI:** 10.3390/cells9071742

**Published:** 2020-07-21

**Authors:** Coralie Croissant, Céline Gounou, Flora Bouvet, Sisareuth Tan, Anthony Bouter

**Affiliations:** Institute of Chemistry and Biology of Membranes and Nano-objects, UMR 5248, CNRS, University of Bordeaux, IPB, F-33600 Pessac, France; coralie33400@free.fr (C.C.); celine.gounou@u-bordeaux.fr (C.G.); flora.bouvet@u-bordeaux.fr (F.B.); s.tan@cbmn.u-bordeaux.fr (S.T.)

**Keywords:** annexin-A6, membrane repair, human skeletal muscle, cap subdomain, fluorescence, electron microscopy, CLEM

## Abstract

Defects in membrane repair contribute to the development of some muscular dystrophies, highlighting the importance to decipher the membrane repair mechanisms in human skeletal muscle. In murine myofibers, the formation of a cap subdomain composed notably by annexins (Anx) is critical for membrane repair. We applied membrane damage by laser ablation to human skeletal muscle cells and assessed the behavior of annexin-A6 (AnxA6) tagged with GFP by correlative light and electron microscopy (CLEM). We show that AnxA6 was recruited to the site of membrane injury within a few seconds after membrane injury. In addition, we show that the deficiency in AnxA6 compromises human sarcolemma repair, demonstrating the crucial role played by AnxA6 in this process. An AnxA6-containing cap-subdomain was formed in damaged human myotubes in about one minute. Through transmission electron microscopy (TEM), we observed that extension of the sarcolemma occurred during membrane resealing, which participated in forming a dense lipid structure in order to plug the hole. By properties of membrane folding and curvature, AnxA6 helped in the formation of this tight structure. The compaction of intracellular membranes—which are used for membrane resealing and engulfed in extensions of the sarcolemma—may also facilitate elimination of the excess of lipid and protein material once cell membrane has been repaired. These data reinforce the role played by AnxA6 and the cap subdomain in membrane repair of skeletal muscle cells.

## 1. Introduction

Plasma membrane of numerous cell types, such as cardiac or skeletal muscle cells during contraction [1,2], epithelial cells during the passage of food bolus [3] or endothelial cells submitted to blood flux [4], endure mechanical stress that can induce membrane damages. For example, it has been observed that up to 20% of skeletal muscle cells can be damaged during physical exercise [1]. Currently, the absence or failure of membrane resealing leads to cell death and may contribute to the development of degenerative diseases, such as muscular dystrophies. Among these, limb-girdle muscular dystrophy type R2 (LGMDR2, formerly 2B) or Miyoshi myopathy and LGMDR12 (formerly 2 L) are, respectively characterized by mutations in the dysferlin and anoctamin-5 genes, leading to several dysfunctions including a failure in cell membrane repair process [5,6].

Following plasma membrane damage, extracellular mM Ca^2+^ concentration enters the cell. The increase of intracellular Ca^2+^ concentration triggers the repair machinery in eukaryotic cells ensuring a rapid resealing of large plasma membrane ruptures [7,8,9]. Several proteins have been identified as belonging to the membrane repair machinery such as AHNAK, acid sphingomyelinase, ESCRT complexes, MG53, S100 proteins, SNAREs, synaptotagmins, calpains, caveolins, dysferlin or Anx [10]. The Anx family consists of twelve soluble proteins in mammals, named AnxA1 to A13 (the number 12 is non-assigned) [11]. Anx share the property of binding to membranes exposing negatively charged phospholipids in a Ca^2+^-dependent manner [11,12], with little specificity for anionic lipid head-groups, yet the Ca^2+^ concentration required for binding varies considerably between Anx [13,14,15]. Anx present a common carboxy-terminal membrane-binding domain formed by the cyclic arrangement of conserved 70 amino-acid repeats [11,16]. This membrane-binding domain has the shape of a slightly curved rhomboid with a convex membrane-binding face where Ca^2+^-binding loops are exposed and a concave face from which protrudes the amino-terminal domain. The amino-terminal part is very variable in size and is assumed to be responsible for the functional specificity of Anx [11,12,16,17]. The presence of different Anx in skeletal muscle [18,19,20,21]—together with the participation of several members of the Anx family in membrane repair processes [22,23]—raises the question of a collective role of these proteins in the protection and repair of sarcolemma injuries.

AnxA6, which is the largest Anx with a molecular weight of 68 kDa, exhibits eight 70 amino-acid conserved sequences forming two modules of four repeats [12]. It has been reported that at high Ca^2+^ concentration (about 2 mM), AnxA6 is able to bind two distinct phosphatidylserine-containing membranes, probably by the two distinct modules [24]. The use of this property for promoting membrane repair remains elusive so far. Implication of AnxA6 in the sarcolemma repair machinery has been mainly characterized in animal models. Roostalu and Strähle have shown that AnxA6 knock-down in muscle cells leads to a form of myopathy due to a defective membrane repair in zebrafish [19]. They have observed that dysferlin and AnxA6 accumulate at the disruption site independently one to each other and are immediately followed by AnxA1 and A2, building a crucial scaffold for membrane repair. The absence of AnxA6 inhibits the accumulation of AnxA1 and A2, indicating that the sequential recruitment of Anx is therefore crucial for membrane repair [19]. The formation of a similar scaffold has been observed in murine myofibers [25]. After membrane injury, AnxA1, A2, A5 and A6 aggregate into a tight structure positioned on the exterior surface of the myofiber that the authors termed the “repair cap” subdomain (hereafter termed cap subdomain). Adjacent to the cap subdomain is a region the authors referred to as the “shoulder” subdomain”—where phosphatidylinositol-4,5-bisphosphate, phosphatidylserine, dysferlin, MG53, BIN1 and EHD2 accumulate [25]. Regarding human cells, the rapid recruitment of Anx in a tight structure, which may look like a cap subdomain, has been observed for AnxA5 in skeletal muscle cells and AnxA4 and AnxA6 in cancer epithelial cells [26,27]. In these cancer epithelial cells, Nylandsted and collaborators have shown that AnxA6 induces membrane curvature, which may facilitate membrane resealing [27,28].

Hitherto, no muscular dystrophy has been linked to AnxA6 deficiency, but the etiology of numerous muscular dystrophies remains unknown. Nevertheless, AnxA6 has been identified as being a genetic modifier of muscular dystrophy [21,29]. Indeed, a truncated form of AnxA6, called AnxA6N32, prevents the translocation of wild-type AnxA6 to the disruption site in mice muscle fibers, leading to a reduced or inexistent cap subdomain and inhibition of membrane repair in these cells. The importance of AnxA6 in membrane repair has been recently reinforced by the demonstration that the treatment with recombinant AnxA6 protects against acute muscle injury in wild-type mice and reduced the level of serum creatinine kinase, a biomarker of disease, in a model of muscular dystrophy [30]. A dysfunction of AnxA6 in human muscle cells may therefore greatly impact the severity of the disease.

Here, we analyzed the response of myotubes established from a human skeletal muscle cell line to membrane damage by laser ablation. We observed that myotubes rendered deficient for AnxA6 suffered from a defect of membrane repair. In damaged myotubes, the behavior of AnxA6 coupled to GFP was analyzed and compared to AnxA5 and dysferlin. We observed that AnxA6 belongs to the cap subdomain built during membrane resealing. By means of CLEM, we characterized the cap subdomain, which is notably composed of an extension of the cell membrane. This extension of the cell membrane may promote membrane resealing and also participate in the compaction of lipid and protein material that has to be eliminated after membrane repair.

## 2. Materials and Methods

### 2.1. Culture of Human Skeletal Muscle Cells

The healthy LHCN-M2 (referred to hereafter as LHCN) cell line was provided by the platform for immortalization of human cells from the Center of Research in Myology (Paris, France). This cell line was established from satellite cells of the pectoralis major muscle of a 41-year-old subject [31]. Myoblasts were cultured in a skeletal muscle medium composed by one volume of Medium 199 with glutaMAX^TM^ (Gibco^®^ by by Thermo Fisher Scientific, Waltham, MA, USA), four volumes of Dubelcco’s modified Eagle Medium (DMEM) with high-glucose and glutaMAX^TM^ and without pyruvate (Gibco^®^ by Thermo Fisher Scientific, Waltham, MA, USA) supplemented with 20% fetal bovine serum, 50-µg/mL gentamycin and a commercial mix of skeletal muscle cell growth medium supplements (ref. C-39365, Promocell, Heidelberg, Germany), which included 12.5-µg/mL fetuin, 2.5-ng/mL human recombinant epidermal growth factor, 0.25-ng/mL basic fibroblast growth factor, 2.5-µg/mL insulin, 0.1-µg/mL dexamethasone. Myotubes were obtained by cultivating 90%-confluence myoblasts for three days in a differentiation medium, composed of the skeletal muscle medium supplemented only with 10-µg/mL insulin.

The MDA-MB-231 cancer cell line was cultured in DMEM containing high-glucose, pyruvate and glutaMAX™ (Gibco^®^ by Thermo Fisher Scientific) supplemented with 10% fetal bovine serum.

### 2.2. Western Blot

Myotubes were trypsinized, pelleted and resuspended in 300 µL of Dulbecco’s phosphate buffer saline (D-PBS) depleted in Ca^2+^ and supplemented with 1-mM EGTA. Sonication of ice-cold cell suspension was performed with a Branson digital sonifier (amplitude 20%, duration 2 min, interval 5 s and pulse 5 s) and two successive centrifugations at 13,000 g for 1 min allowed to remove cell debris. 10 μg protein extracts were separated on 10% SDS-PAGE. Tank electrophoretic transfer (Bio-Rad, Hercules, CA, USA) onto PVDF membrane was performed for 1 h at 100 V. AnxA5 (35 kDa) and AnxA6 (68 kDa) were, respectively detected with mouse anti-AnxA5 (Sigma, Saint-Louis, MI, USA) and anti-A6 (Santa Cruz Biotechnology, Dallas, TX, USA) monoclonal antibodies and GAPDH (loading control) was detected with a rabbit anti-GAPDH polyclonal antibody (Santa Cruz Biotechnology). Primary antibodies were diluted 1:1000 in saturation solution composed by Tris buffer saline (10-mM Tris, 150-mM NaCl, pH 8.0) supplemented with 0.1% Tween20 and 5% non-fat dry milk. Revelation was performed using secondary antibodies conjugated to horse-radish peroxidase (GE-Healthcare, Chicago, IL, USA) diluted 1:2000 in saturation solution and Opti-4CN™ colorimetric kit (Bio-Rad, Hercules, CA, USA). ImageJ software was used to measure the relative intensity of protein bands.

### 2.3. Membrane Rupture and Repair Assay

Membrane repair was assayed as recently reported [32], according to a protocol that differed slightly from our previous studies [26,33]. Myotubes were cultured in complete growth medium on 18*18-mm glass coverslips (Nunc). A solution of 5-µg/mL FM1-43 (Thermo Fisher Scientific, Waltham, MA, USA) in D-PBS containing 2-mM Ca^2+^ was maintained over ice and subsequently added in a homemade coverslip cell chamber, where the coverslip was mounted. To induce membrane damage, cells were irradiated at 820 nm with a tunable pulsed depletion laser Mai Tai HP (Spectra-Physics, Irvine, USA) of an upright two-photon confocal scanning microscope (TCS SP5, Leica, Wetzlar, Germany) equipped with an HCX APO L U–V-I 63.0 × 0.90 water lens. Irradiation consisted of 1 scan (1.3 s) of a 1 µm × 1 µm area with a power of 110 (±5) mW. 512 × 512 images were acquired at 1.3 s intervals with pinhole set to 1 Airy unit. Membrane rupture and repair processes were monitored by measuring variations in fluorescence intensity of FM1-43 as previously described [26,33]. The FM1-43 was excited by the 488-nm laser line (intensity set at 30% of maximal power) and fluorescence emission was measured between 520 nm and 650 nm. For quantitative analysis, the fluorescence intensity was integrated over the whole cell surface and corrected for the fluorescence value recorded before irradiation, using ImageJ software.

### 2.4. Transduction of AnxA6-Targetting shRNA Lentiviral Particles in LHCN Myotubes

The following shRNA sequences, cloned into the pLKO.1 puro-vector (MISSION^®^ shRNA plasmids, Sigma, Saint-Louis, MI, USA), were used: AnxA6-targetting shRNAA6-1 (TRCN0000011461, Sigma): ^5′^CCGGCGG GCACTTCTGCCAAGAAATCTCGAGATTTCTTGGCAGAAGTGCCCGTTTTT^3′^; shRNAA6-2 (TRC N0000008686, Sigma): ^5′^CCGGCGGTTGGTGTTCGATGAGTATCTCGAGATACTCATCGAACACCAACCGTTTTT^3′^; control shRNA: ^5′^CCTAAGGTTAAGTCGCCCTCGCTCGAGCGAGGGCGACTTAACCTTAGG^3′^. The two AnxA6-targetting shRNAs were selected from a commercial library according to the “mean knockdown level” given by the supplier (0.9 and 0.87, respectively). Lentiviral-based particles containing shRNAs were produced by Bordeaux University lentiviral vectorology platform (US005, Bordeaux, France) by transient transfection of 293T cells. MDA-MB-231 cells and LHCN myoblasts were cultured in their respective growth medium for 24 h. Transduction was carried out by adding concentrated lentiviral particles to the cells at a multiplicity of infection (MOI) of 100 in Opti-MEM^®^ medium for 24 h. Cells were subsequently incubated with growth medium for 24 h and then 2-µg/mL puromycin (Sigma, Saint-Louis, MI, USA) were added in order to select transduced cells. 72 h after antibiotic treatment, western-blot analysis or immunocytofluorescence experiments were performed as described in Section 2.2 and Section 2.6, respectively. To establish Anx-deficient myotubes, myoblasts were incubated in differentiation medium for 7 h 30 and then transduced with shRNA lentiviral particles at a MOI of 100 in Opti-MEM^®^ medium for 24 h. Subsequently, cells were incubated in differentiation medium for 24 h and 2-µg/mL puromycin were added for 72 h in order to select transduced myotubes.

### 2.5. Subcellular Traffic of AnxA6-GFP in Damaged Myotubes

The pA6-GFP plasmid was constructed by cloning AnxA6 cDNA into the pEGFP-N1 (Takara Bio USA, Mountain View, CA, USA) plasmid and was a gift of Volker Gerke (University of Munster, Germany). The pA5-mCh plasmid, which contained recombinant AnxA5-mCherry, was constructed from pA6-GFP using the In-Fusion^®^ HD Cloning kit (Takara Bio USA, Mountain View, CA, USA). Anx-GFP or Anx-mCherry fusion proteins harbored the fluorescent tag at the C-terminus. Myotubes at 65 h of differentiation were transfected using Lipofectamine 2000 reagent (Thermo Fisher Scientific, Waltham, MA, USA). 0.4 µg plasmid was mixed with 0.52 µL Lipofectamine 2000 for 20 min at room temperature and the mixture was incubated with cells cultured on 18*18-mm glass coverslip for 4 h at 37 °C in 200 µL Opti-MEM^®^ (Thermo Fisher Scientific, Waltham, MA, USA). Transfection was renewed once and myotubes were then incubated for at least 24 h in differentiation medium before analysis. For myotubes co-expressing pA6-GFP together with pA5-mCh, fluorescent signal of GFP and mCherry is, respectively presented in green and magenta within the manuscript, for a better visualization of AnxA5-mCherry. Magenta is the color that the human eye best distinguishes from green [34].

### 2.6. Immunocytofluorescence

Cells were fixed and permeabilized in 100% methanol solution (5 min, at −20 °C). All subsequent steps (antibody incubation and washes) were performed using 2% BSA in D-PBS solution. Primary antibody at 1:100 and secondary antibody at 1:1000 were successively incubated with cells for 1 h at 37 °C. Antibodies were mouse monoclonal anti-AnxA6 antibody (Santa Cruz Biotechnology, sc-271859), mouse monoclonal anti-Dysferlin antibody (Leica, NCL-Hamlet), Alexa Fluor 488-coupled anti-mouse goat antibody and Alexa Fluor 546-coupled anti-rabbit donkey antibody (Thermo Fisher Scientific, Waltham, MA, USA). Finally, cells were washed in D-PBS and nuclear counterstaining was performed with DAPI (Sigma, Saint-Louis, MI, USA).

### 2.7. Immuno-TEM

For subcellular localization of endogenous Anx and CLEM in damaged cells, myotubes were formed and cultured in a 35-mm glass bottom dish equipped with a square-patterned coverslip (MatTek, Ashland, MA, USA). Cell membrane rupture was performed by laser ablation according to the protocol described above, but in the absence of FM1-43 to avoid fluorescence crosstalk. Myotubes were fixed in 1% glutaraldehyde solution for 20 min at room temperature, as described previously [35]. They were subsequently incubated in 25-mM ammonium chloride for 15 min and permeabilization and saturation of nonspecific sites were performed in a mixture of 2% BSA and 0.1% TritonX100 in D-PBS for 30 min. All subsequent steps (antibody incubation and washes) were performed using 2% BSA in D-PBS solution. Mouse anti-AnxA6 monoclonal antibody at 1:100 and secondary Alexa Fluor 488- and gold nanoparticles-conjugated anti-mouse goat antibody (FluoroNanogold, Nanoprobes, NY, USA) at 1:100 were successively incubated with myotubes for 1 h at 37 °C. After three rinses, myotubes were observed by fluorescence microscopy. Cells were then postfixed overnight at 4 °C in a mixture of 4% paraformaldehyde and 2% glutaraldehyde in 0.1-M cacodylate buffer (pH 7.4). Signal amplification was performed using the HQ silver kit (Nanoprobes, NY, USA) according to the manufacturer’s instructions. Cells were treated with 1% osmium tetroxide in 0.1-M cacodylate buffer for 1 h at room temperature and then dehydrated with ethanol and finally embedded in Epon-Araldite. Thin sections (65 nm) were collected using EM UC7 ultramicrotome (Leica, Wetzlar, Germany) and stained successively with 5% uranyl acetate and 1% lead citrate. TEM observation was performed with a FEI CM120 operated at 120 kV. Images were recorded with a USC1000 slow scan CCD camera (Gatan, Pleasanton, CA, USA).

## 3. Results

### 3.1. Expression and Distribution of AnxA6 in Human Skeletal Muscle Cells

**Figure 1:**
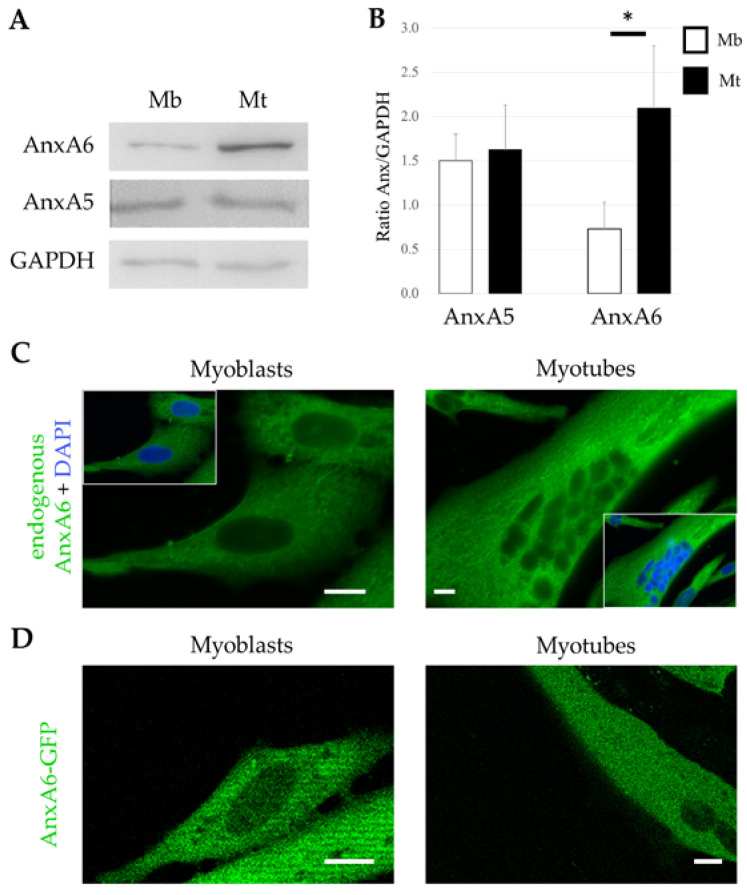
Expression and subcellular distribution of AnxA6 in human myoblasts and myotubes. (**A**,**B**) Cellular content in endogenous AnxA5 and AnxA6 in healthy LHCN-M2 (LHCN) myoblasts (Mb) and myotubes (Mt) was quantified through western-blot analysis; (B) data are mean ± SEM from five experiments. Wilcoxon test, * *p* < 0.05; (**C**) subcellular localization of endogenous AnxA6 (green) in LHCN myoblasts and myotubes by immunocytofluorescence. In the insets, nuclear counterstaining with DAPI is displayed (blue). Scale bars: 10 µm; (**D**) subcellular localization of AnxA6-GFP in living LHCN myoblasts and myotubes by fluorescence microscopy. Scale bars: 10 µm.

In order to investigate the relative expression of AnxA6 in human skeletal muscle cells, western-blot analysis was performed from the healthy human myogenic cell line LHCN, which was established from satellite cells isolated from pectoralis major adult muscle [31]. We observed that myotubes showed a 3-fold increase in AnxA6 expression compared to myoblasts (Figure 1A,B), whereas AnxA5 expression is unchanged between myoblasts and myotubes as previously reported [26]. By immunocytofluorescence, we observed that endogenous AnxA6 is exclusively cytoplasmic in LHCN myoblasts and myotubes (Figure 1C). Homogenous distribution of the fluorescence suggested that AnxA6 is mainly cytosolic. When LHCN myoblasts and myotubes were transfected by an AnxA6-GFP expression plasmid, we observed that the fusion protein also exhibited a cytosolic localization (Figure 1D).

We concluded therefore that AnxA6 localizes exclusively in the cytoplasm of human myoblasts and myotubes, as previously described for other cell types or species [19,36]. AnxA6 expression is enhanced in myotubes, which suggests it is important for physiological processes in differentiated muscle cells.

### 3.2. AnxA6 is Mandatory for Membrane Repair in Human Skeletal Muscle Cells

In order to study the involvement of AnxA6 in membrane repair, we had to generate AnxA6-deficient myotubes. As with other post-mitotic cells, transfection or transduction of differentiated muscle cells remains a challenging task. Here, we applied a shRNA strategy as previously performed for the knock-down of AnxA5 in LHCN myotubes [26]. Screening experiments were performed using the MDA-MB-231 cell line, which is easier to be transduced, with two candidate shRNAs selected from a commercial library. Western-blot analysis showed that both shRNAs are able to specifically reduce AnxA6 expression of more than 90% systematically (n = 3, Figure S1). Using experimental conditions established with MDA-MB-231 cells, LHCN myoblasts were then transduced with both shRNAs. AnxA6 expression in transduced LHCN myoblasts was about 40% lower than in control cells, in a fairly similar way whatever the shRNA sequence (Figure S2). No synergy effect was observed when both shRNA sequences were mixed. As expected, the level of knock-down was lower in myoblasts compared to MDA-MB-231 cells. LHCN myotubes, which are multinucleated cells measuring several hundred µm long, are normally established from fusion of myoblasts cultured three days in the differentiation medium [26] (Figure S3A). However, as previously reported for AnxA5-deficient LHCN myoblasts [26], we observed that LHCN myoblasts rendered deficient in AnxA6 were unable to form myotubes (Figure S3B), suggesting that AnxA6 is involved in the process of cell differentiation and/or fusion. This result also implied that shRNA transduction had to be carried out during the formation of myotubes. We determined that transduction had to be performed 8 h after starting incubation of myoblasts in differentiation medium. Since western-blot analysis requires a large number of cells and prevents to distinguish between myotubes and myoblasts remaining in culture, we quantified the effect of shRNAA6 transduction specifically in myotubes by immunocytofluorescence. Preliminary experiments showed that quantification by immunocytofluorescence gave similar results to western-blot analysis regarding the relative expression of AnxA6 in shRNA-transduced or control LHCN myoblasts (Figure S4). Whatever the shRNA sequence used; we observed a decrease of about 60% of the expression of AnxA6 in shRNA-transduced LHCN myotubes (Figure S5). Subsequent experiments were performed using the LHCN myotubes transduced with the shRNAA6-2 sequence, which are hereafter named AnxA6-deficient myotubes.

**Figure 2:**
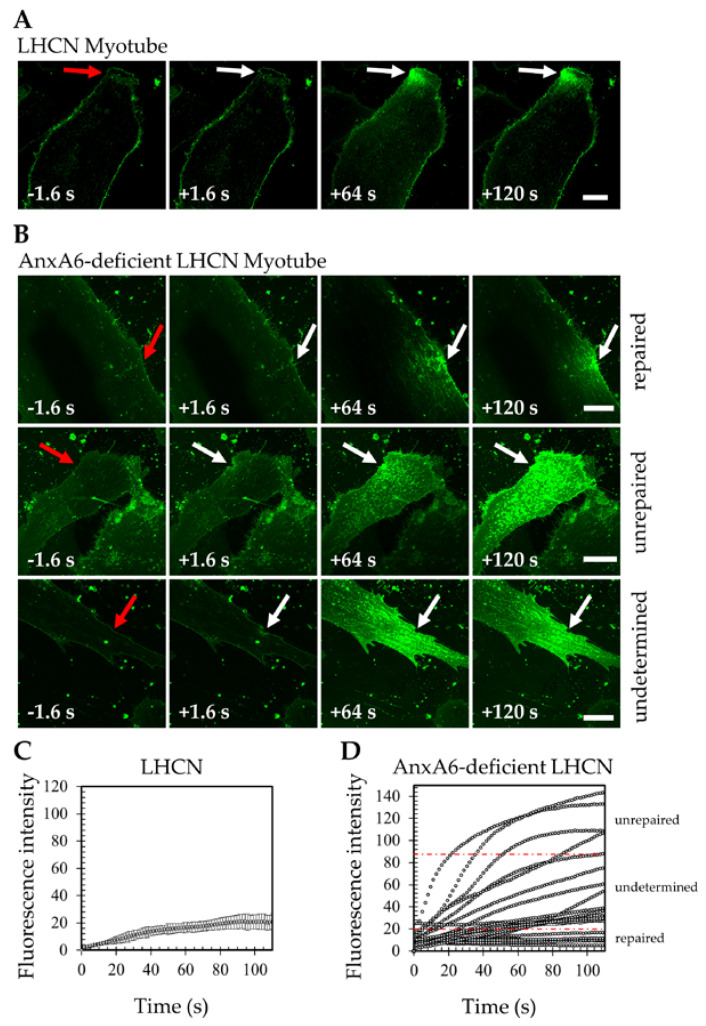
Responses of AnxA6-deficient LHCN myotubes to laser ablation. (A-B) Sequences of images showing the response of a wild-type (**A**) and three different AnxA6-deficient (**B**) LHCN myotubes to 110-mW laser irradiation in the presence of 2-mM Ca^2+^ and FM1-43 (green). In these panels, the area of membrane irradiation is marked with a red arrow before irradiation and a white arrow after irradiation. Time at which the image was recorded before (-) or after (+) irradiation is indicated. Scale bars = 20 µm; (**C**,**D**) Data represent the FM1-43 fluorescence intensity integrated over whole cell section, which is plotted versus time. For LHCN myotubes (mean ± SEM of 20 cells within 2 independent experiments), the fluorescence intensity reached a plateau after ~80 s (**C**). For AnxA6-deficient myotubes, very different kinetics were observed from one myotube to another. As an example, one typical experiment with 20 cells is presented in (**D**). When the presence of a plateau and a maximal fluorescence intensity lower than 20 a.u. were observed, the cell was classified as “repaired cell” (lower part of the graph, as indicated). When the kinetics of fluorescence changes showed a continuous increase with values higher than 90 a.u., the cell was classified as “unrepaired cell” (upper part). Otherwise, cells were classified as “undetermined cells”. Data analysis is presented in Table S1. Red dashed lines divide the three types of response.

**Figure 3:**
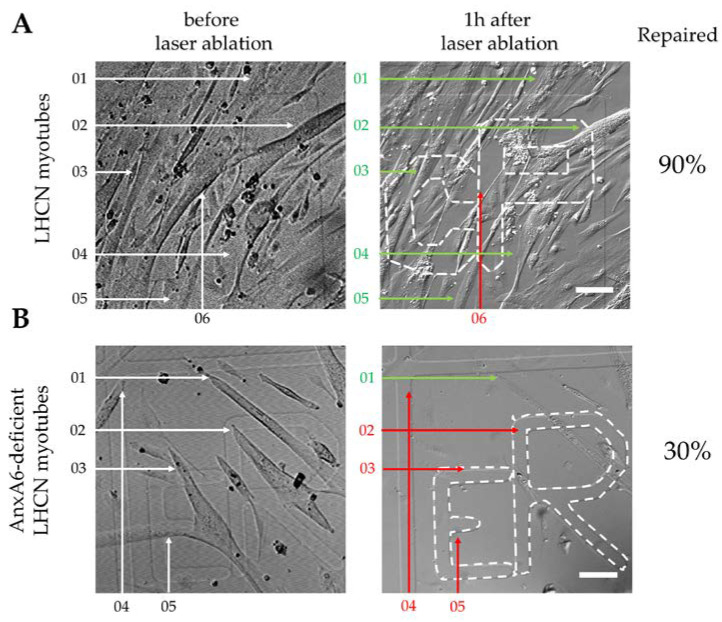
Laser ablation leads to the death of AnxA6-deficient LHCN myotubes. (**A**) Control or (**B**) AnxA6-deficient LHCN myotubes were cultivated on a square-patterned coverslip displaying an alphanumeric code that enabled cell tracking. Left-hand images exhibit myotubes before laser ablation. Each arrow indicates the area to be damaged, which is identified by a number. On the right-hand images, the alphanumeric code has been drawn for sake of clarity. Green arrows indicate myotubes that are still present 1 h after laser ablation. In contrast, red arrows point out myotubes that disappeared. About 100 cells over 5 independent experiments were analyzed for each condition. Data analysis is presented in Table S2. Scale bars = 100 µm.

Sarcolemma repair assay was performed by laser ablation in the presence of Ca^2+^ and FM1-43, as previously described [32,33]. By analyzing changes in the FM1-43 intracellular fluorescence intensity, we first confirmed that LHCN myotubes are able to reseal a µm-size membrane damage in about 80 s (Figure 2A,C) as previously reported [26]. When AnxA6-deficient LHCN myotubes were submitted to the same irradiation conditions, different types of response were observed. Some myotubes exhibited an increase in fluorescence intensity limited to an area close to the disruption site (Figure 2B, repaired). The kinetics of fluorescence intensity changes were characterized by an increase for about 70–90 s and then the presence of a plateau (Figure 2D, repaired), which indicated a rapid resealing of cell membrane. Some other myotubes exhibited a strong and large increase of the intracellular fluorescence intensity (Figure 2B,D, unrepaired), which meant that they were unable to reseal cell membrane damage. Finally, for a significant part (about 50%) of them we were unable to determine whether cell membrane resealing occurred or not (Table S1). Indeed, kinetics associated with these myotubes exhibited whether a weak, but continuous raise of the intracellular fluorescence intensity or the presence of a plateau at a very high level of fluorescence intensity (Figure 2B,D undetermined). These complex and unclear responses of AnxA6-deficient myotubes to laser membrane damage result probably from a variable AnxA6 expression due to an uncomplete shRNA knock-down. We had therefore to slightly modify the way to analyze membrane repair. Control and AnxA6-deficient myotubes were cultured on glass bottom dishes equipped with a square-patterned coverslip that enabled cell tracking during different stages of the experiment (Figure 3). A field identified by means of the alphanumeric code was imaged and myotubes present in the field were successively damaged by laser ablation exactly as in the standard membrane repair assay. In the case of control LHCN myotubes, which are able to rapidly reseal sarcolemma damage, we observed that a large majority (about 90%, Table S2A) of damaged myotubes were still adhered on the coverslip 1 h after laser ablation (Figure 3A). A similar result was observed when LHCN myotubes were transduced with a scrambled shRNA (data not shown). However, when AnxA6-deficient LHCN myotubes were submitted to the same experimental conditions, we observed that only 30% of myotubes were still present on the coverslip 1 h after laser ablation (Figure 3B and Table S2B). It is likely that the loss of 70% of myotubes is due to the absence of membrane repair, which leads to the death of cells that are released from the coverslip. We therefore concluded that the deficiency in AnxA6 compromises sarcolemma repair, suggesting that AnxA6 is a crucial component of the membrane repair machinery in human skeletal muscle cells.

### 3.3. Recruitment of AnxA6 at the Membrane Disruption Site and Formation of the Cap Subdomain

All proteins composing the repair machinery, notably dysferlin [5], Anx [18,19,37,38,39] and MG-53 [40], are rapidly recruited to the membrane disruption site. In order to investigate the behavior of AnxA6 during human sarcolemma injury, we analyzed the subcellular trafficking of AnxA6 fused to GFP in laser-damaged LHCN myotubes. For this purpose, laser ablation was performed at two different positions on cells in order to study the kinetics of recruitment from two different perspectives. Membrane damage was carried out either at the lateral edge —which is the conventional way to perform membrane damage by laser ablation—or on the surface of the myotube (Figure S6). Irradiation at the edge of the cell allowed to visualize the wounded area sideways, whereas the irradiation on the surface allowed a view from the top.

**Figure 4:**
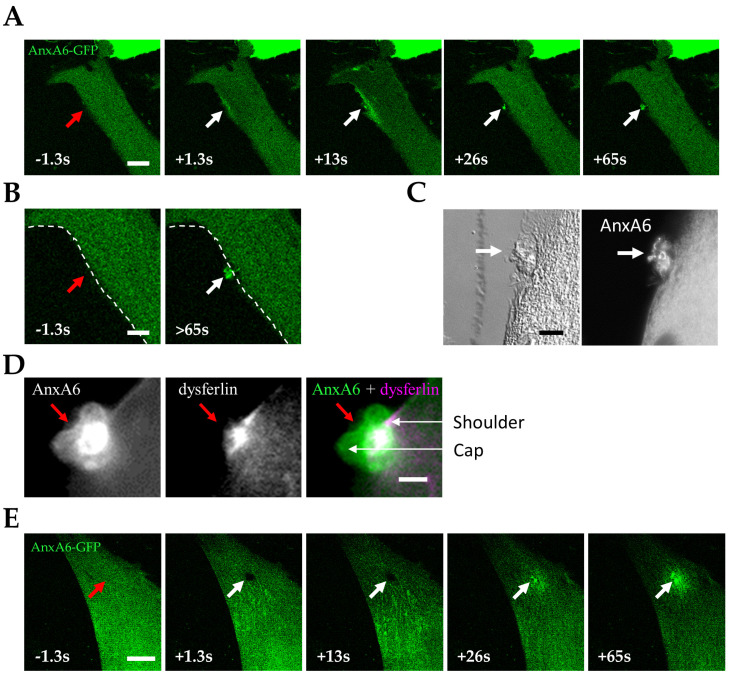
Recruitment of AnxA6 to the damaged membrane site and formation of the cap subdomain in LHCN myotube. (**A**) LHCN myotubes were transfected with the plasmid pA6-GFP and membrane damage experiments were performed by laser ablation at the lateral edge of the myotube. Red arrow, area before irradiation; white arrow, area after irradiation. Scale bar: 10 µm; (**B**) Magnified images of the disruption site before (left) and more than 65 s (right) after laser ablation. The dashed white line corresponds to the position of the plasma membrane before irradiation. Scale bar: 5 µm; (**C**) Distribution of endogenous AnxA6 in a damaged LHCN myotube. The plasma membrane of LHCN myotubes was damaged by laser irradiation. Myotubes were immunostained for AnxA6 and observed in bright-field (left) and fluorescence microscopy (right). White arrow, irradiated area. Scale bar: 5 µm; (**D**) Distribution of AnxA6 and dysferlin between cap and shoulder subdomains. A LHCN myotube expressing AnxA6-GFP (green) was damaged at the lateral edge and then fixed and immunostained for dysferlin (magenta). Red arrow, irradiated area (**E**) LHCN myotubes were transfected with the plasmid pA6-GFP and membrane damage experiments were performed by laser ablation on the surface of the myotube. Red arrow, area before irradiation; white arrow, area after irradiation. Scale bar: 20 µm.

We observed that AnxA6-GFP was recruited to the disruption site immediately after membrane damage and appeared to be primarily associated with the plasma membrane (Figure 4A, +1.3 s and Video S1). Fluorescence intensity increased mainly at the plasma membrane around the rupture site over a large area (+13.0 s). From about 20 s after membrane injury, AnxA6-GFP that was interacting with the plasma membrane seemed to be released from the inner leaflet and went back to the cytoplasm (Video S1). This result suggested that intracellular Ca^2+^ concentration rapidly decreased, probably while plasma membrane was resealed. Then, we observed that AnxA6-GFP concentrated in a structure (+26.0 s) that persisted beyond 65 s (Figure 4B). We checked by immunocytofluorescence that endogenous AnxA6 accumulated similarly in damaged control LHCN myotubes (Figure 4C). This tight structure that was positioned on the exterior surface of the myotube is identical to the cap subdomain previously described in murine myofibers [25]. In these cells, adjacent to the cap subdomain is the shoulder subdomain, which is composed notably by dysferlin, MG53, BIN1 and EHD2 [25]. To confirm the presence of both subdomains in repaired LHCN myotubes, we damaged AnxA6-GFP-expressing myotubes by laser ablation and performed immunostaining of dysferlin. If we observed a region where AnxA6 and dysferlin overlapped, we were able also to distinguish two specific regions with either AnxA6-GFP or dysferlin corresponding, respectively to the cap and shoulder subdomains (Figure 4D). When laser ablation was performed on the surface of the myotube, we observed a slower recruitment of AnxA6-GFP to the disruption site (Figure 4E and Video S2). For instance, no increase of fluorescence intensity was observed around the disruption site within the first 20 s after membrane damage. This slower dynamic resulted probably from a stronger membrane damage, as the laser penetrated deeply in the cytoplasm. AnxA6-GFP was finally recruited about 20 s after membrane injury in a wave-like motion (Video S2). Such a behavior, which was not identified when the wounded area was visualized sideways, has been previously reported for S100A11 during its recruitment to the laser-injured membrane site in HeLa cells [41]. Molecular mechanisms responsible for this specific motion remain to be elucidated. After about 30 s, we observed that AnxA6-GFP accumulated at the disruption site, plugging the hole created by the membrane injury (Figure 4E). A strictly different behavior was observed in damaged MCF-7 cancer cells, in which AnxA6-GFP is recruited to wound edges where it promotes the closure of the hole by triggering contraction of the membrane edges [27]. The role played by AnxA6 in the processes of membrane repair may therefore be different in human skeletal muscle and cancer epithelial cells.

**Figure 5:**
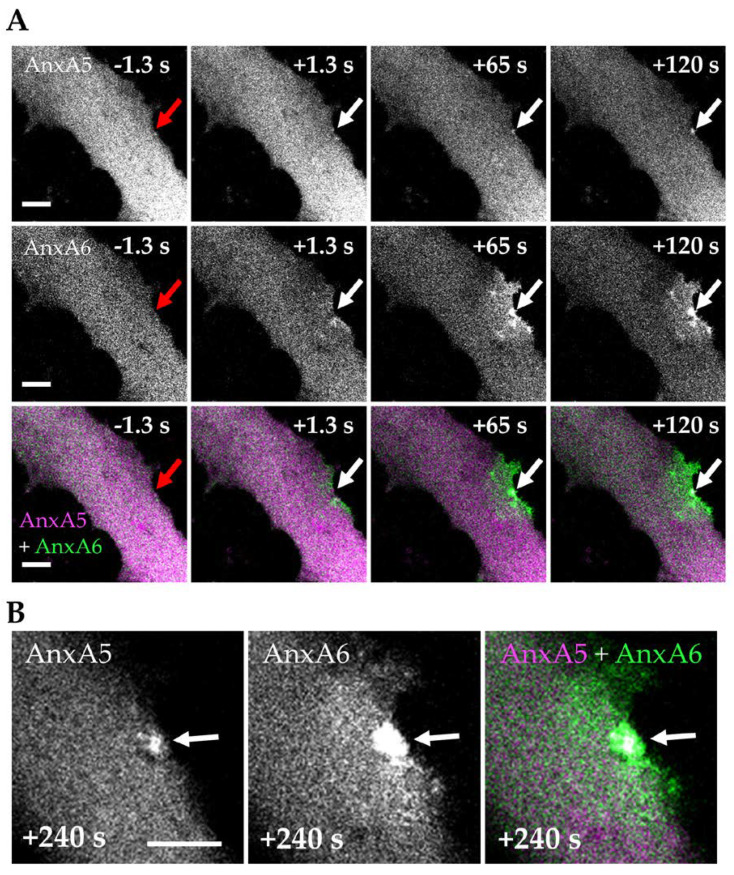
Distribution of AnxA5 and AnxA6 within the cap subdomain. LHCN myotubes were transfected with the plasmids pA6-GFP and pA5-mCherry to express AnxA6-GFP (green) and AnxA5-mCherry (magenta) and membrane damage experiments were performed by laser ablation at the edge of the myotube. (**A**) Follow-up of the recruitment of AnxA6 and AnxA5 within 120 s after membrane damage. Red arrow, area before irradiation; white arrow, area after irradiation; (**B**) Enlarged images of the damaged area 240 s after membrane damage. Scale bars: 10 µm.

We previously reported that AnxA5 participates in the membrane repair of human skeletal myotubes [26] and AnxA5 has also been observed as composing the cap subdomain in murine myofibers [25]. In order to compare the traffic of AnxA5 and AnxA6 in human skeletal muscle cells during sarcolemma repair, we imaged AnxA5-mCherry and AnxA6-GFP in LHCN myotubes damaged by laser ablation. We observed that AnxA5-mCherry and AnxA6-GFP were recruited nearly immediately and simultaneously to the damaged site of the plasma membrane (Figure 5A and Video S3). Nevertheless, a major difference distinguished AnxA5 from AnxA6: AnxA5 was present in a limited area at the disruption site, while AnxA6 spread more widely on the plasma membrane around the disruption site. AnxA5 and AnxA6 were present in the cap subdomain, AnxA5 composing the core of the subdomain (Figure 5B). This result is consistent with previous studies showing the presence of AnxA5 in the cap subdomain of murine myofibers [25] and human myotubes [26]. We previously showed that AnxA5 forms two-dimensional arrays around the disruption site to prevent the expansion of the tear and promote membrane repair [26,37]. The observation of a small cluster of AnxA5-mCherry in the cap subdomain could correspond to AnxA5 arrays that interacted with the edges of the torn membrane and were then packaged in the cap after membrane resealing. All together, these results indicated that membrane repair in human skeletal muscle cells is based on the formation of a cap subdomain, composed notably by AnxA6.

### 3.4. Visualization of AnxA6 and the Cap Subdomain by TEM

**Figure 6:**
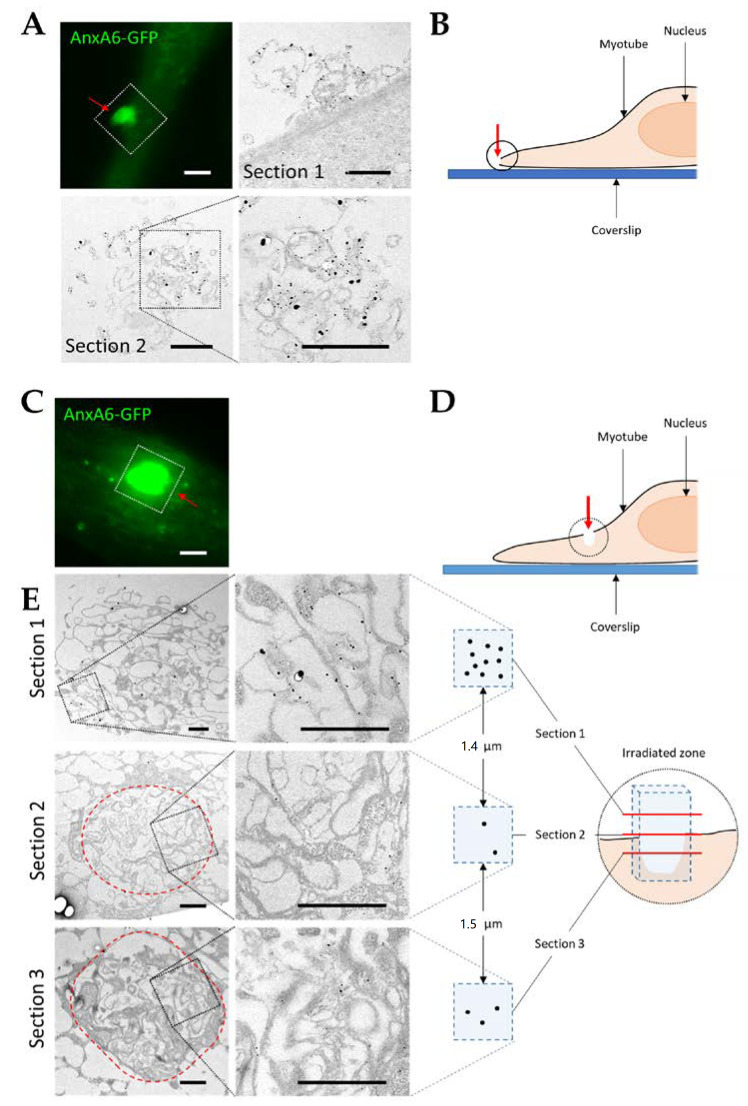
Correlative light and electron microscopy (CLEM) imaging of damaged AnxA6-GFP-expressing LHCN myotubes. LHCN myotubes were transfected with pA6-GFP plasmid and membrane damage was performed on the edge (**A**) or on the surface (**C**, **E**) of the myotubes as featured in (**B**) and (**D**), respectively. After membrane injury, AnxA6-GFP (green) was observed in fluorescence microscopy for about two minutes and chemically fixed. Myotubes were then immunostained for AnxA6 with a secondary antibody coupled with a gold nanoparticle (black particles in **A** and **E**) and analyzed by TEM (bright images in **A** and **E**). Fluorescence images in (**A**) and (**C**) correspond to the localization of AnxA6-GFP about two minutes after membrane injury. In (**E**), the diagram on the right represents the irradiated area and the location of the three sections observed. Red arrow, irradiated area; red dotted lines, boundaries of the damaged area. Scale bar in fluorescence microscopy: 5 µm. Scale bar in TEM: 1 µm.

To further characterize the cap subdomain and the role played by AnxA6 in this structure, we imaged by CLEM damaged AnxA6-GFP-expressing myotubes, as previously described [26]. Briefly, we observed AnxA6-GFP in a single damaged myotube successively through fluorescence microscopy and TEM. Immunostaining of AnxA6 using a secondary antibody coupled to gold nanoparticles enabled to analyze the distribution of the protein in the damaged myotube at high resolution. We first carried out membrane damage at the edge of the myotube (Figure 6A,B) and focused our attention on the characterization of the ultrastructure of the cap subdomain. Indeed, little information was known about the origin, structure and function of the cap subdomain. It has been proposed that the cap subdomain may correspond to an ordered protein scaffold required for membrane repair, which is composed notably by Anx [25]. Even if the cap subdomain can vary considerably in size, TEM images revealed that it is characterized by an accumulation of disorganized membrane material on the exterior surface of the myotube, exactly where laser ablation was performed (Figure 6A and Figure S7). In most cases, membrane material that was accumulated in the cap subdomain appeared as a tangle of filaments, suggesting it is of cell membrane origin. This excess of plasma membrane may arise from lysosome exocytosis, which would increase the membrane surface and thus reduce the tension exerted by the cortical cytoskeleton, as previously proposed [42]. AnxA6-GFP appeared to be accumulated mainly outside the cell in the cap subdomain, interacting primarily with membrane filaments that may correspond to extensions of the sarcolemma (Figure 6A). Endogenous AnxA6 exhibited a similar distribution (Figure S7).

When LHCN myotubes were damaged on their surface, accumulation of membrane material at the disruption site was visible on several sections (Figure 6E). A tangle of membranes was observed in sections located above the plane of the damaged sarcolemma, which may correspond to the cap subdomain that is localized outside the cell (Figure 6E, Section 1). Deeply in the cytoplasm we observed that an accumulation of membrane material filled the entire area damaged by laser ablation. We can hypothesize that the laser penetrated deeply in the cytoplasm and created a significant membrane damage, which required greater mobilization of intracellular material to plug the tear (Figure 6E, Section 2 and Section 3). AnxA6-GFP was found mainly associated with membranes present above the plane of the initial position of the sarcolemma (Section 1) and rarely in cytoplasmic sections (Section 2 and Section 3). AnxA6 seems therefore accumulated essentially in the cap subdomain outside the damaged area, in agreement with the fluorescence images of AnxA6-GFP (Figure 4A).

**Figure 7:**
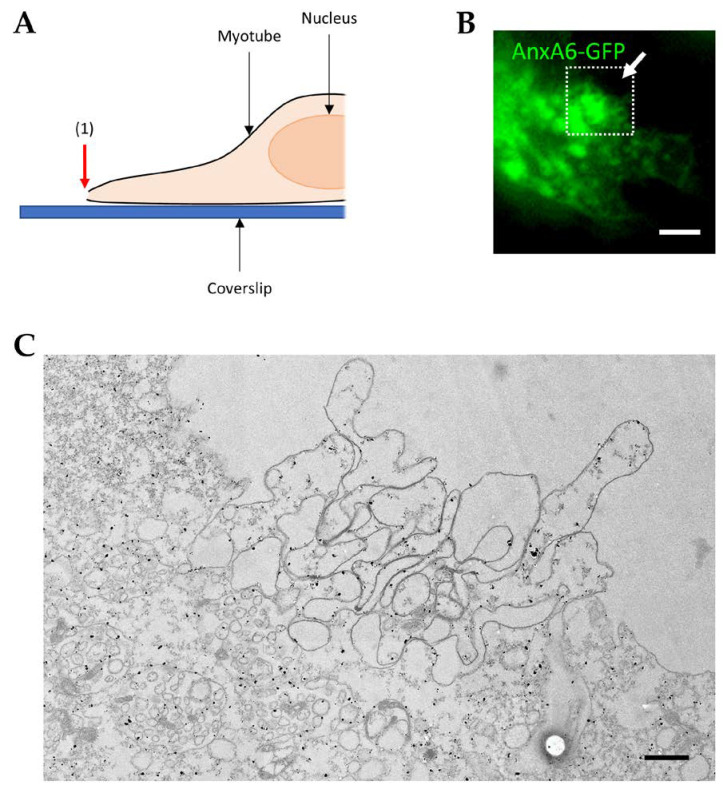
CLEM imaging of AnxA6-GFP-expressing LHCN myotubes during the formation of the cap subdomain. (**A**) LHCN myotubes were transfected with pA6-GFP plasmid and membrane damage was performed at the edge of the myotubes, which were then rapidly fixed. (**B**) Recruitment of AnxA6-GFP was checked by fluorescence microscopy as described in the legend of Figure 6. (**C**) Myotubes were then immunostained for AnxA6 with a secondary antibody coupled with a gold nanoparticle and observed in TEM. Red and white arrows, irradiated area. Scale bar in fluorescence microscopy: 5 µm. Scale bar in TEM: 1 µm.

To get further insight in the formation of the cap subdomain, we sought to fix myotubes rapidly after membrane damage. We had the opportunity to observe the membrane disruption site during the formation of the cap subdomain (Figure 7). This event was characterized by the fact that AnxA6 was still interacting with a large area around the disruption site (Figure 7B).

The typical image displayed in Figure 7C shows the complex structure of the disruption site during membrane resealing (Figure 7C). Multiple intracellular vesicles were present at the base of the membrane material accumulated at the disruption site, supporting the hypothesis of the formation of a lipid patch. We also observed a tangle of membrane filaments that formed the main part of the protrusion. AnxA6 was mainly interacting with these membrane filaments, which obviously looked like extensions of the sarcolemma.

## 4. Discussion

Here we show that the deficiency of AnxA6 in human myotubes compromises sarcolemma repair. This result confirms previous studies that have shown the crucial role played by AnxA6 in membrane repair [19,25,27,30]. We observed that AnxA6 expression increases during differentiation of myoblasts into myotubes, which correlates with the fact that myotubes are more prone to membrane damage and must be well-equipped to cope with more frequent mechanical stress due to contraction. In laser damaged human myotubes, AnxA6 is recruited to the disruption site within seconds after sarcolemma rupture, where it mainly interacts with cell membrane. About one minute after injury, AnxA6 is found in a very tight structure outside the myotube, which is identical to the cap subdomain previously described by McNally and collaborators [25]. The presence of dysferlin at the base of the cap subdomain indicates the existence of shoulder subdomains in human resealing sarcolemma, as described in murine myofibers [25].

Structure, origin and specific function of the cap subdomain remained unclear. It has been reported that it may correspond to a protein scaffold mainly constituted of Anx and essential for membrane repair [25]. Currently, our TEM images reveal the presence of a dense and disorganized membrane structure outside the myotube several minutes after membrane resealing, which corresponds to the cap subdomain. The cap subdomain would therefore not be a protein scaffold that participates in membrane repair, but rather the excess of lipid and protein material to be eliminated after membrane repair. Our TEM images also reveal that during the process of membrane resealing a large part of the membrane material is constituted of “filaments” that seem to correspond to extension of the cell membrane. Expansion of cell membrane triggered by lysosome exocytosis has been already proposed in order to reduce tension exerted by cortical cytoskeleton and thus promote membrane repair [42]. Our finding has resolved a longstanding mystery about “filaments” stained by FM1-43 and randomly observed by fluorescence microscopy during our membrane repair assays. This applied in particular to murine perivascular cells damaged by laser ablation, when the protocol used a powerful laser (160 mW) irradiating a large area (9 µm²) (Video S4) [37].

In collaboration with the cluster of intracellular vesicles, the extension of the cell membrane may participate in the resealing process by forming a dense lipid structure plugging the hole. Compaction of the membrane material in this tangle of cell membrane may also facilitate its elimination once cell membrane was resealed. In zebrafish myofibers, it was observed that this task is performed by macrophages [43]. This would explain why the cap subdomain persists for several tens of minutes after membrane injury in our experiments, where only muscle cells are present.

Numerous studies question the existence of the lipid “patch” for membrane-resealing. We hypothesize that the formation of a lipid “patch” [8] and the cap subdomain are not mutually exclusive and may coexist. They could actually correspond to two successive stages of the membrane repair process, the formation of the cap subdomain resulting from the excess of membranes produced for membrane resealing and intended to be released.

Which is the role of AnxA6 in these processes? During membrane resealing, we observed that AnxA6 interacts essentially with plasma membrane. With AnxA5, AnxA6 is part of less Ca^2+^-sensitive Anx for membrane binding [44]. The Ca^2+^ gradient created at the disruption site after membrane injury supports the binding of AnxA6 to cell membrane rather than to intracellular vesicles present deeply in the cytoplasm. It has been proposed that AnxA6 induces a constriction force closing the tear after AnxA4-induced plasma-membrane invagination in damaged MCF-7 cells [27]. Unlike MCF-7 cells, no inward invagination was observed at the disruption site in damaged human skeletal muscle cells. In addition, when membrane damage was performed on the surface of the myotube, neither fluorescence video-microscopy nor TEM images revealed the presence of AnxA6 surrounding the hole created by membrane disruption. AnxA6 may therefore act differently in cancer epithelial MCF-7 and muscle LHCN cells. It is important to note that AnxA4 is highly expressed in cancer cells [45,46] and that MCF-7 cells were exposed to strong injuries by laser irradiation inducing a large wound diameter (up to 3 μm) in the study cited above [27]. As the membrane repair mechanism may vary depending on the cell type, the extent of the damage and the spatial position of the injury [23,27,47], different experimental conditions may explain different behaviors observed for AnxA6.

The Anx family has been shown to be able to modify membrane morphology by inducing curvature and folding [27,28]. Together with their ability to aggregate adjacent membranes [14,24], Anx could allow the compaction of extended cell membrane present at the disruption site in order to promote membrane resealing and then to facilitate the release of the membrane excess. We have shown that AnxA6 is rapidly recruited to the disruption site, by mainly interacting with the plasma membrane. At the disruption site, the excess of plasma membrane covered by AnxA6 could initiate the formation of the cap subdomain. By inducing folding and curvature of the extensions of cell membrane, AnxA6 may allow to form a tight structure plugging the hole. Once sarcolemma was resealed, AnxA6 is exclusively found in the cap subdomain interacting with accumulated membranes. It may therefore help at condensing membranes to be eliminated, this elimination being probably performed by macrophages [43]. The presence of two Anx core domains gives the ability to AnxA6 to bridge two adjacent membranes [24], such as two regions of the cell membrane and/or vesicle membranes. In addition, phylogenetic analysis of Anx has revealed that the core domain present at the N-terminus was similar to AnxA3 whereas at the C-terminus it was similar to AnxA5 [48]. Anx induce different and specific morphologies upon interaction with membranes [28]. For instance, AnxA3 rolls the membrane in a fragmented manner from free edges, producing multiple thin rolls, whereas AnxA5 induces cooperative roll-up of the membrane. AnxA6 may therefore induce multiple membrane rearrangements at the disruption site, which promote membrane repair and the formation of the cap subdomain.

**Figure 8:**
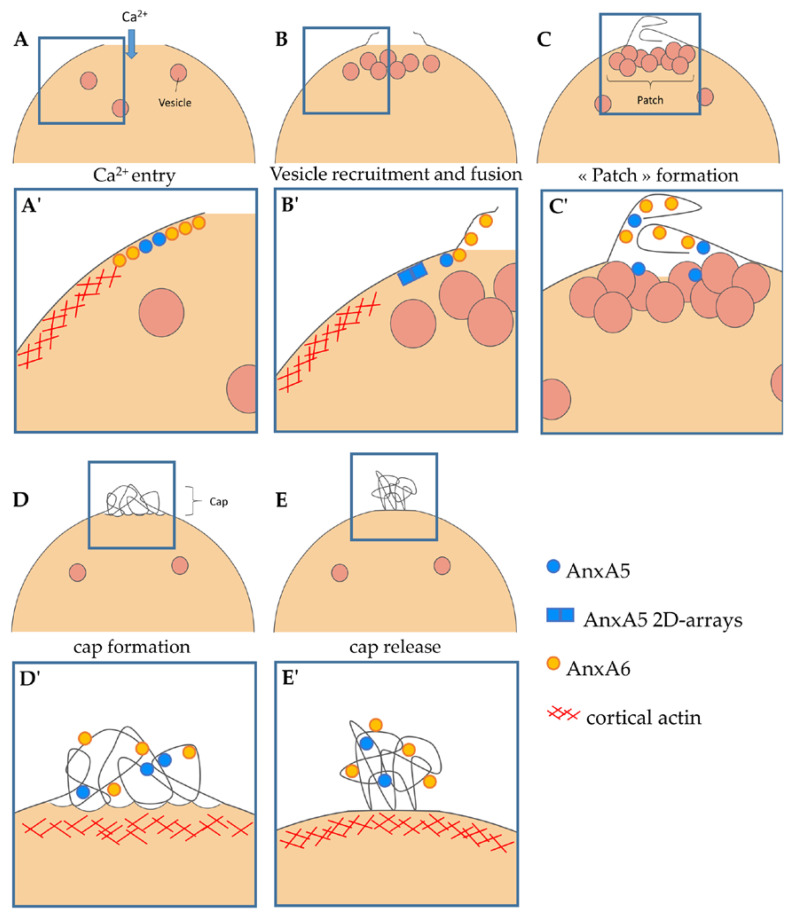
Model of membrane repair in human skeletal muscle cells. (**A**,**A’**) Entry of Ca^2+^ induces the recruitment of Anx to the plasma membrane, notably AnxA5 and AnxA6. (**B**,**B’**) The membrane tension is reduced by depolymerization of actin and exocytosis of lysosomes [42]. The increase in sarcolemma surface leads to excess membrane at the disruption site on which AnxA6 is associated. AnxA5 forms 2D arrays that strengthen the sarcolemma and limit the expansion of the tear, as previously reported [26]. Intracellular vesicles are recruited to the disruption site. (**C**,**C’**) Aggregation of intracellular vesicles forms a “patch” that plugs the rupture. AnxA6 and maybe AnxA5, induce the folding of the extensions of sarcolemma in order to form a tight structure. (**D**,**D’**) Accumulation of Anx leads to the folding and curvature [28] of membranes and the formation of the cap subdomain. (**E**,**E’**) The integrity of the sarcolemma is restored by the newformed plasma membrane and the elimination of the cap subdomain by macrophages (not represented) [43].

Taken together our results enable to propose a model of the implication of AnxA6 in membrane repair of human skeletal muscle cells (Figure 8). The entry of Ca^2+^ induces the binding of AnxA6 to membranes surrounding the disruption site (Figure 8A,A’). At this stage, depolymerization of cortical actin and lysosome exocytosis induced by the massive increase of intracellular Ca^2+^ concentration may lead to a reduction in the tension exerted by the cortical cytoskeleton, as proposed by Togo et al. [42]. Increase in membrane surface by lysosome exocytosis may lead in the formation of an extension of sarcolemma at the disruption site, on which AnxA6 is attached (Figure 8B,B’). At this time, AnxA5 forms 2D arrays at the edges of the torn sarcolemma to prevent the expansion of the tear, as previously reported [26]. The aggregation of intracellular vesicles recruited to the disruption site may then form a lipid patch in order to plug the hole and interrupt exchanges between the intra- and extracellular environments (Figure 8C,C’). Anx and particularly AnxA6, may induce folding and curvature [28] of the excess of sarcolemma, thus initiating the formation of a tight membrane structure. Membrane excess due to lengthened sarcolemma and input of intracellular vesicles may form a structure where Anx accumulate (Figure 8D,D’). The ability of Anx to induce membrane aggregation and curvature [28] allows the compaction of this membrane excess to form the cap subdomain. The newformed membrane and the elimination of the cap subdomain by other cell types such as macrophages [43], may allow the membrane integrity of the cell to be regained (Figure 8E,E’).

## Supplementary Materials

The following are available online at https://www.mdpi.com/2073-4409/9/7/1742/s1, Figure S1: Decrease in the expression of endogenous AnxA6 in MDA-MB-231 cells by shRNA strategy, Figure S2: Decrease in the expression of endogenous AnxA6 in shRNA-transduced LHCN myoblasts, Figure S3: The absence of differentiation and/or fusion of AnxA6-deficient LHCN myoblasts, Figure S4: Knock-down of AnxA6 in LHCN myoblasts assessed by immunocytofluorescence, Figure S5: Knock-down of AnxA6 in LHCN myotubes by shRNA strategy, Figure S6: Schematic representation of the positioning of laser for membrane injury on myotubes, Figure S7: Characterization of the ultrastructure of the cap subdomain, Table S1: Data analysis of the responses of AnxA6-deficient LHCN myotubes to laser ablation, Table S2: Data analysis of the responses of AnxA6-deficient LHCN myotubes to laser ablation according to the protocol presented in Figure 3, Video S1: Behavior of AnxA6-GFP in response to membrane damage by laser ablation performed at the lateral edge of a LHCN myotube, Video S2: Behavior of AnxA6-GFP in response to membrane damage by laser ablation performed on the surface of a LHCN myotube, Video S3: Behavior of AnxA6-GFP and AnxA5-mCherry in response to membrane damage by laser ablation in a LHCN myotube, Video S4: Membrane filaments emerge from murine perivascular cell damaged by powerful laser irradiation.
