# Supplementary material for: Annexin-A6 in Membrane Repair of Human Skeletal Muscle Cell: A Role in the Cap Subdomain
Source: Cells. 2020 Jul 21;9(7):1742. doi: 10.3390/cells9071742 (PMC7409186; doi:10.3390/cells9071742)
Supplement: Supplementary file 1 [file cells-09-01742-s001.zip › Supp_Material_Bouter/SupplementalFigure_MuscleRepair_A6_Bouter_Final.pdf]

cells

MDPI

Type of the Paper Article

Annexin-A6 in membrane repair of human skeletal muscle cells: a role in the cap subdomain

Coralie Croissant, Céline Gounou, Flora Bouvet, Sisareuth Tan, and Anthony Bouter*

Institute of Chemistry and Biology of Membranes and Nano-objects, UMR 5248, CNRS, University of Bordeaux, IPB, F-33600 Pessac, France. coralie33400@free.fr (C.C.); celine.gounou@u-bordeaux.fr (C.G.); flora.bouvet@u-bordeaux.fr (F.B.); s.tan@cbmn.u-bordeaux.fr (S.T.).

* Correspondence: anthony.bouter@u-bordeaux.fr (A.B.).

Received: date; Accepted: date; Published: date

SUPPLEMENTAL DATA

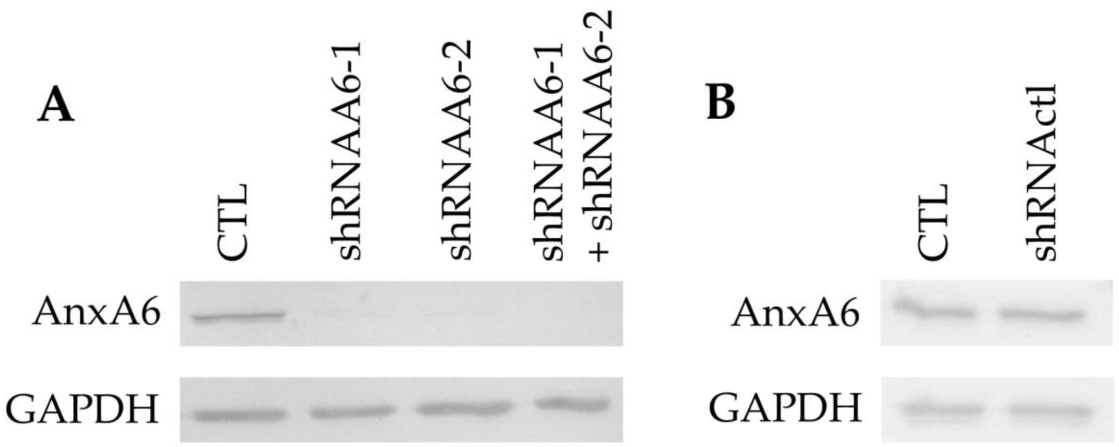

Figure S1. Decrease in the expression of endogenous AnxA6 in MDA-MB-231 cells by shRNA strategy. (A) We have tested two AnxA6-targetting shRNAs from a commercial library (Sigma) according to the "Mean KnockDown Level" given by the purchaser. MDA-MB-231 cells were transduced with shRNAA6-1, shRNAA6-2 or both mixed shRNAA6-1 and shRNAA6-2 sequences at a final MOI of 100. Non-transduced cells (CTL) were used as control. The cellular content of AnxA6 was quantified by western-blot using an SDS-PAGE using primary mouse monoclonal anti-AnxA6 and rabbit polyclonal anti-GAPDH (loading control) antibodies. Secondary antibodies were coupled with horse radish peroxidase, which was revealed by colorimetric detection. Results showed great effectiveness of shRNA sequences in decreasing the expression of AnxA6, with a decrease of more than 90%. (B) We also performed western-blot analysis of MDA-MB-231 cells transduced with a control scrambled shRNA (shRNActl) that did not recognize any mRNA sequences in humans, and no difference was observed between control and non-transduced cells (CTL). From these first series of experiments we have concluded that our protocol was operational and both shRNAs are functional whether they are used alone or in combination.

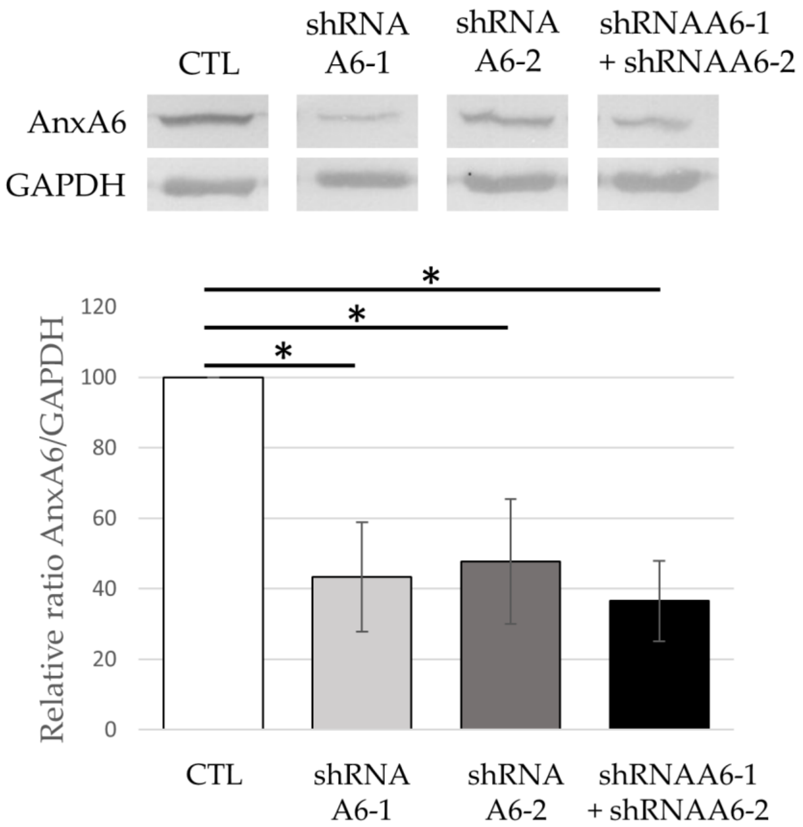

Figure S2. Decrease in the expression of endogenous AnxA6 in shRNA-transduced LHCN myoblasts. LHCN myoblasts were transduced at a MOI of 100 with shRNAA6-1, shRNAA6-2 or shRNAA6-1 + shRNAA6-2 sequences. Non-transduced cells (CTL) were used as control. The cellular content of AnxA6 in myoblasts was quantified by western-blot as described in Figure S1. ANOVA one-factor test, *: $p<0.05$.

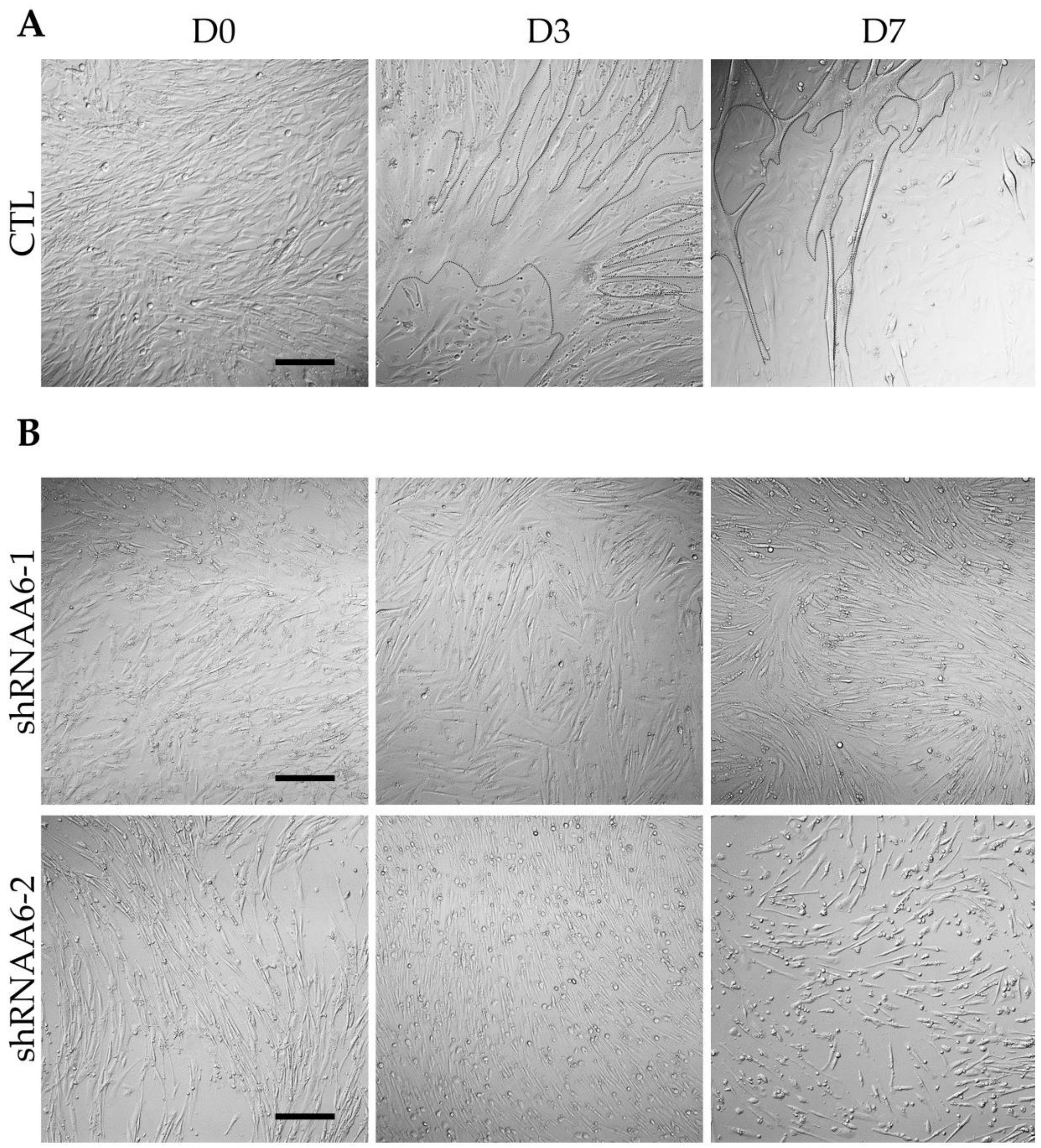

Figure S3. The absence of differentiation and/or fusion of AnxA6-deficient LHCN myoblasts. (A) LHCN myoblasts were incubated in the differentiation medium for 5 days. D0 is the day when myoblasts at a confluence of about 90% were incubated in the differentiation medium. At D3 and D7, cell membrane of myotubes was drawn for sake of clarity. (B) LHCN transduced with shRNAA6-1 or shRNAA6-2 were treated as described in (A). We observed that transduced myoblasts were unable to form myotubes. Scale bar: 200 μm.

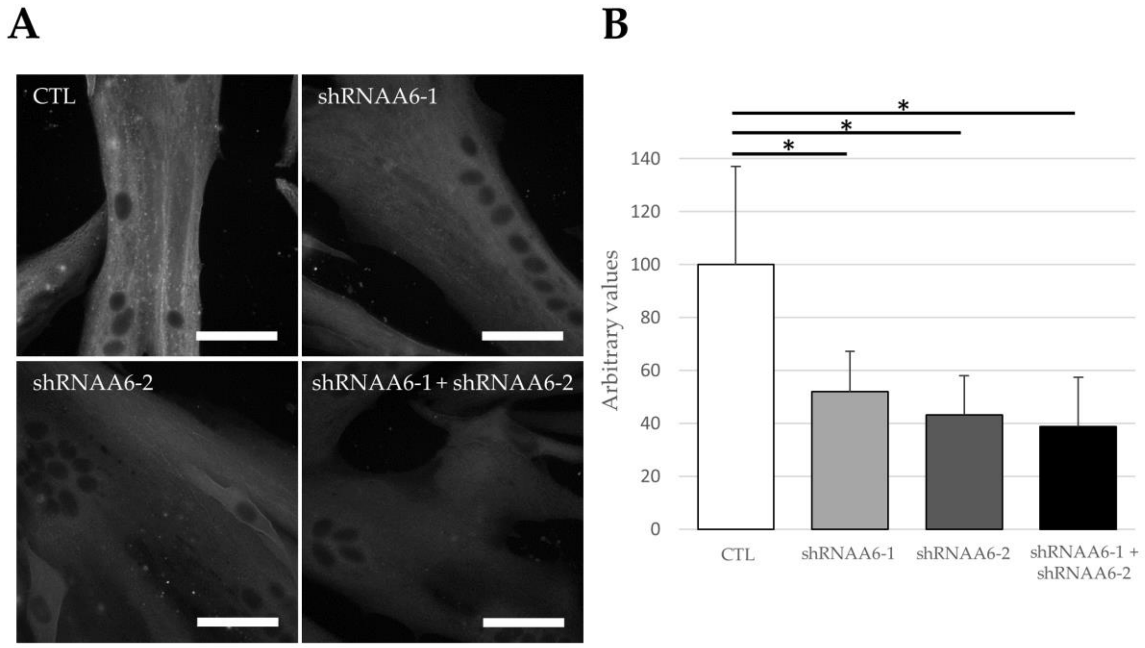

Figure S4. Knock-down of AnxA6 in LHCN myotubes by shRNA strategy. (A) Immunocytofluorescence analysis of the expression of endogenous AnxA6 in transduced and non-transduced (CTL) LHCN myotubes. Transduction was performed by adding shRNA-containing lentiviral particles 7h30 after starting the incubation of LHCN myoblasts in the differentiation medium. (B) Fluorescence intensity was analyzed with the ImageJ software. Kruskal-Wallis test *: $p<0.05$. Scale bar: 40 μm.

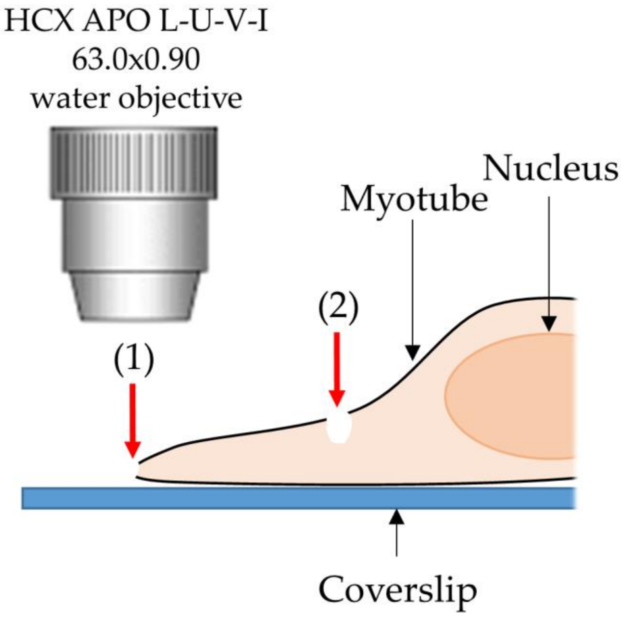

Figure S5. Schematic representation of the positioning of laser for membrane injury on myotubes. Irradiation was carried out from the top of the cell with an upright microscope either on the lateral edge of the myotube (1) or on the surface of the myotube (2).

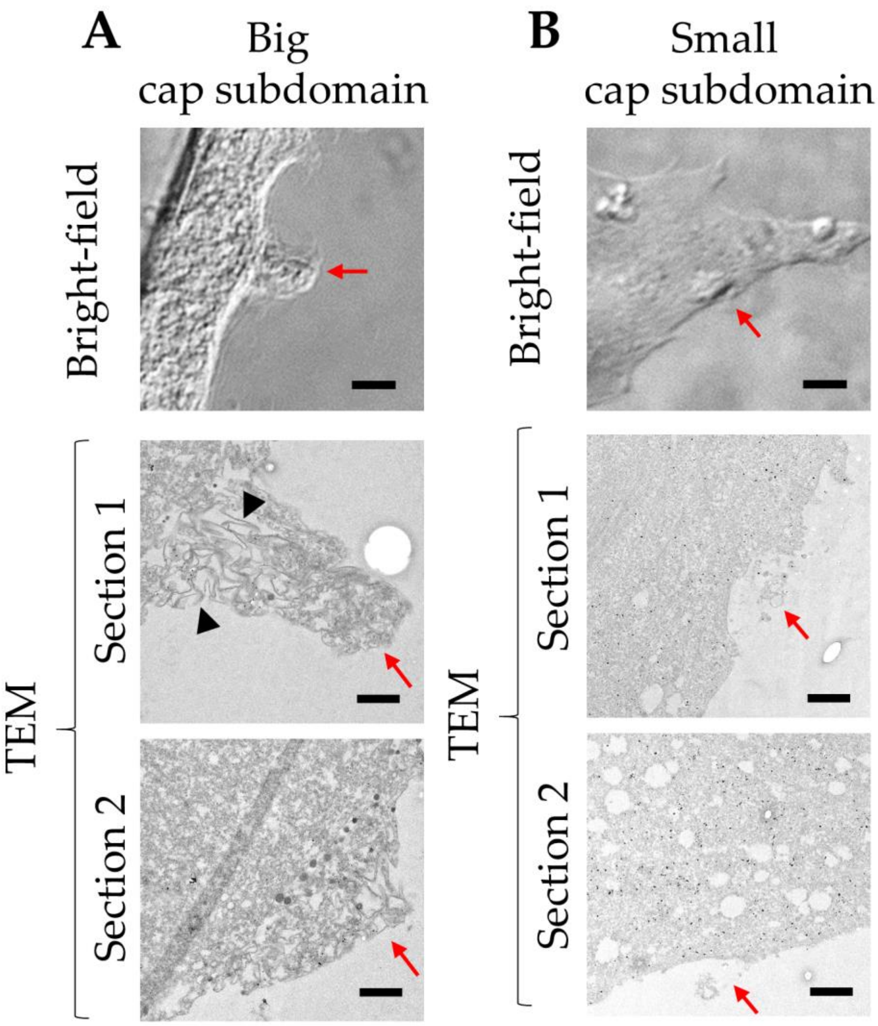

Figure S6. Characterization of the ultrastructure of the cap subdomain. LHCN myotubes were damaged by laser ablation, fixed and immunostained for AnxA6 and then observed by TEM. Two typical series of images displaying either a big (A) or a small (B) cap subdomain were presented. The upper image was acquired by bright-field microscopy. The two bottom images were obtained by TEM and represent two different sections of the resin block. Red arrows and black arrowheads point out respectively the cap subdomain and extensions of the sarcolemma.

SUPPLEMENTAL VIDEOS

Video S1 shows the behavior of AnxA6-GFP in response to membrane damage by laser ablation performed at the lateral edge of a LHCN myotube. See the legend of Figure 4A. Frame rate = 5 fps.

Video S2 shows the behavior of AnxA6-GFP in response to membrane damage by laser ablation performed on the surface of a LHCN myotube. See the legend of Figure 4E. Frame rate = 5 fps.

Video S3 shows the behavior of AnxA6-GFP and AnxA5-mCherry in response to membrane damage by laser ablation in a LHCN myotube. See the legend of Figure 5. Frame rate = 5 fps.

Video S4 presents a murine perivascular cell damaged by laser irradiation consisted of three successive scans of a 3×3-µm area with a power of 160 mW in the presence of Ca^{2+} and FM1-43 (white). This protocol was used in our early experiments in membrane repair of murine perivascular cells in 2010 [39]. From the disruption site emerged some kind of filaments that rippled in the extracellular medium. As the filaments are stained with FM1-43, it is likely that they correspond to cell membrane. Scale: field width = 78 µm. Frame rate = 5 fps.
